# Heart Block and Sinus Pause Following Abdominal Surgery: A Case Requiring Temporary Pacemaker Insertion

**DOI:** 10.7759/cureus.40964

**Published:** 2023-06-26

**Authors:** Muhammad Ghallab, Muhammad Haseeb ul Rasool, Daniel Miller, Mahmoud Alashry, Nicole C Noff, Asma U Hosna, Giovina Collura

**Affiliations:** 1 Internal Medicine, Icahn School of Medicine at Mount Sinai, New York City (NYC) Health and Hospitals, New York, USA; 2 Internal Medicine, Icahn School of Medicine at Mount Sinai, Queens Hospital Center, New York, USA; 3 Cardiology, Icahn School of Medicine at Mount Sinai, New York City (NYC) Health and Hospitals, New York, USA

**Keywords:** sinus node dysfunction, cardiology, abdominal surgery complications, pacemaker, sinus pause

## Abstract

This case report presents the clinical course of a 70-year-old female with a history of hypertension who developed sinus pauses following abdominal surgery, ultimately requiring the placement of a pacemaker. The patient initially presented with altered mental status preceded by abdominal pain, which progressed to confusion and obtundation. Examination revealed signs of toxicity, tachycardia, tachypnea, and a distended abdomen with absent bowel sounds. A computed tomography (CT) scan of the abdomen indicated closed-loop small bowel obstruction with free air and ascites. The patient underwent exploratory laparotomy, revealing purulent fluid and a necrotic, perforated appendix, leading to appendectomy and peritoneal irrigation. Subsequent surgeries addressed the coagulative necrosis of the omentum and wound closure. During the recovery period, the patient exhibited bradycardia with sinus pauses, including episodes of complete heart block. Cardiology consultation attributed this to increased parasympathetic tone following surgery and recommended the placement of a temporary transvenous pacemaker. As the patient's condition improved, the sinus pacing function progressively returned, leading to the removal of the pacemaker. This case underscores the potential development of sinus pauses after abdominal surgery and highlights the importance of prompt recognition, appropriate management, and collaboration between surgical and cardiology teams to ensure patient recovery.

## Introduction

Sinus pauses, also known as sinus node dysfunction, refer to a delay in generating or propagating the electrical current within the sinus node of the heart. These pauses can temporarily cease the heartbeat and are typically characterized by an absence of P-waves on an electrocardiogram (EKG) [1]. Surgery is a major stressor on the body. One potential complication of abdominal surgery is the development of bradycardia, a condition characterized by an abnormally slow heart rate and even sinus pauses [2]. There are multiple factors that can attribute to sinus pause or heart block following surgery. These may include cytokine production or autonomic dysregulation from manipulating the abdominal cavity [3].

In this case report, we present a 70-year-old female patient who underwent abdominal surgery and subsequently developed sinus pauses and heart block during the recovery period. Prompt recognition and appropriate management were crucial in ensuring the patient's recovery. Collaboration between surgical, intensive care, and cardiology teams played a pivotal role in providing comprehensive care and achieving optimal outcomes.

## Case presentation

We present the case of a 70-year-old female with a known medical history of hypertension who was brought to the emergency department by emergency medical service (EMS) due to altered mental status preceded by abdominal pain. According to the patient's family, she had been in her usual state of health until four days before presentation when she began experiencing lower abdominal pain after consuming food from an outside source. Despite taking ibuprofen for pain relief, she only experienced minimal improvement. Two days later, she developed dark-colored stools and progressive confusion on the day of admission. Despite taking ibuprofen for pain relief, she only experienced minimal improvement.

Upon arrival, the patient appeared toxic, exhibiting signs of distress, including restlessness, non-responsiveness, tachycardia, and tachypnea. Her mucus membranes were dry, and a physical examination revealed a distended and tense abdomen with no audible bowel movements. The surgical team promptly requested a computed tomography (CT) scan of the abdomen, which showed a dilated loop of small bowel in the abdomen with a transition point in the pelvis, indicative of closed-loop small bowel obstruction. The CT scan also revealed the presence of free air around the small bowel loop and mild complex ascites. Furthermore, large mesenteric and retroperitoneal lymph nodes were scattered throughout the abdominal region, suggesting possible lymph node involvement.

Given the suspected perforated viscus causing peritonitis, the patient was immediately taken to the operating room for an exploratory laparotomy while undergoing resuscitation. During the procedure, approximately 800 ml of purulent fluid was observed upon opening the peritoneum and multiple pockets of frank pus. The peritoneal cavity was thoroughly irrigated with abundant saline. Upon further examination of the bowel, the appendix was found to be necrotic and perforated, necessitating an appendectomy. The gut was subsequently returned to the peritoneal cavity, and the abdomen was closed using a wound vacuum device with plans for a second-look laparotomy later.

Postoperatively, the patient was difficult to extubate, due to apnea when the ventilator support was temporarily withheld, leading to her transfer to the ICU, where she was intubated and placed on mechanical ventilation. Additionally, she required intravenous (IV) pressors due to hemodynamic instability. The patient remained febrile and septic, necessitating treatment with broad-spectrum intravenous antibiotics and the use of a cooling blanket to manage the elevated temperature. A second laparotomy revealed coagulative necrosis of the omentum and multiple fibrinous adhesions. The necrotic omentum was resected, and adhesiolysis was performed. However, the closure of the abdomen was not feasible due to tension, resulting in the placement of a wound vacuum.

**Figure 1:**
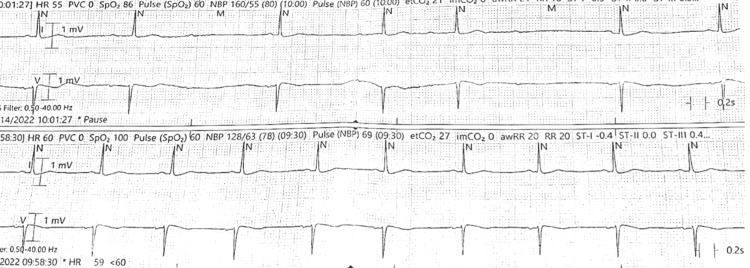
EKG rhythm strips show normal sinus rhythm in the lower strip and bradycardia with Mobitz type I second-degree heart block in the upper strip EKG: electrocardiogram

**Figure 2:**
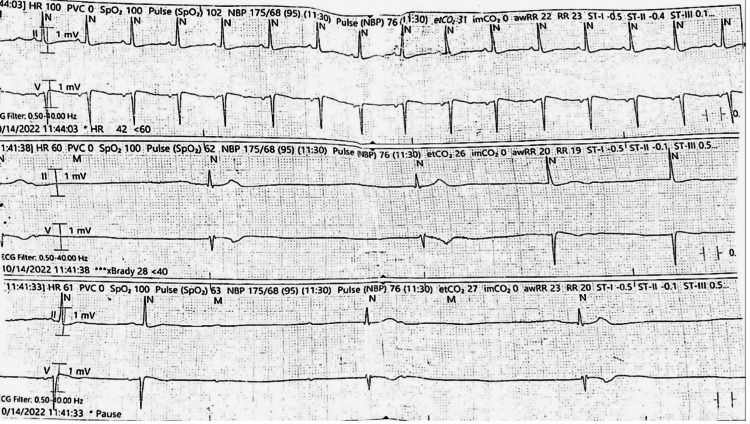
EKG rhythm strips show sinus tachycardia in the upper strip, sinus arrest in the middle strip with escape junctional rhythm, and sinus arrest with complete heart block in the lower strip EKG: electrocardiogram

**Figure 3:**
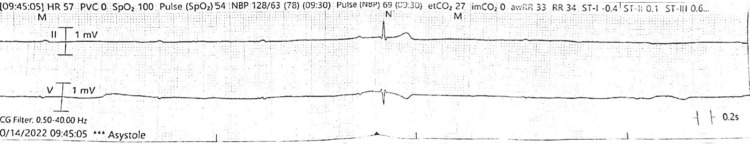
EKG rhythm strip shows complete heart block and sinus arrest with junctional escape beat EKG: electrocardiogram

**Figure 4:**
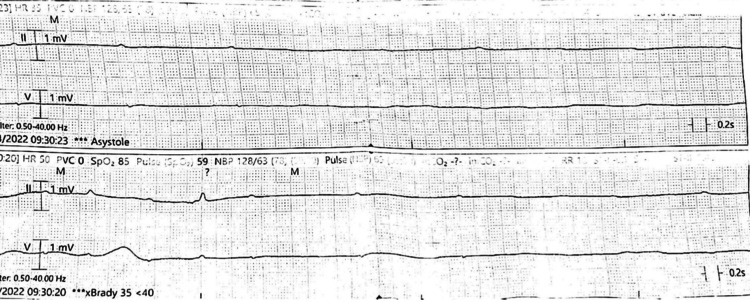
EKG rhythm strips show severe bradycardia and prolonged pauses with complete heart block with no ventricular escape (asystole) in the upper strip and occasional escape beats in the lower strip EKG: electrocardiogram

As the patient's condition gradually improved, she was successfully weaned off pressor support and began the progressive reduction of sedatives, including propofol, fentanyl, and dexmedetomidine. However, during the weaning process, she developed bradycardia with Mobitz type I second-degree heart block (Figure 1), sinus arrest, and complete heart block with no ventricular escape characterized by pauses of 9-14 seconds (Figures 2-4).

**Figure 5:**
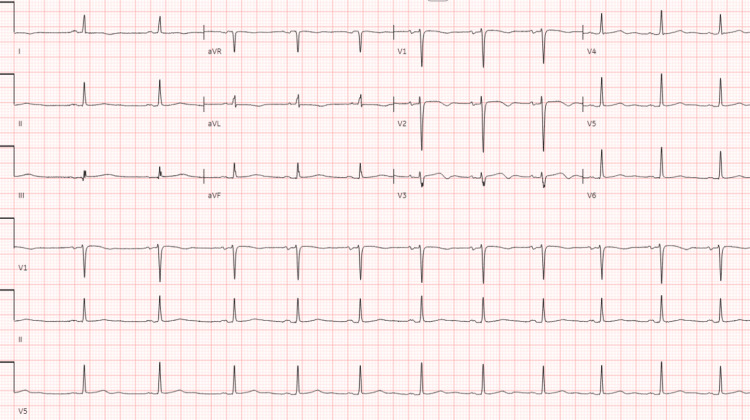
A 12-lead EKG showing normal sinus rhythm EKG: electrocardiogram

Cardiology service was consulted, and a temporary transvenous pacemaker was recommended due to the high-degree atrioventricular (AV) block, believed to be caused by increased parasympathetic tone following surgery. After stabilizing the patient, the pacing rate was adjusted to 40 beats per minute. An echocardiogram revealed preserved ventricular function without any intraventricular thrombus. As intra-abdominal pressure decreased during the recovery period, the patient's sinus pacing function gradually returned; eventually, the transvenous pacemaker was removed. Normal sinus rhythm was noted on EKG subsequently (Figure 5).

Continued care for the patient was provided by the surgical ICU team, where her condition deteriorated due to pneumonia, which progressed to sepsis and then septic shock, and the patient finally deceased after a prolonged hospital course.

## Discussion

Sinus pauses, also known as sinus node dysfunction, refer to a delay in the generation or propagation of the electrical current within the sinus node of the heart. These pauses can lead to a temporary cessation of the heartbeat and are typically characterized by an absence of P-waves on an electrocardiogram (EKG). While sinus pauses can occur spontaneously even in healthy individuals [1], this discussion focuses on their development following abdominal surgery that required a pacemaker.

Abdominal surgery is a major surgical procedure that involves significant physiological stress and may result in various complications. Postoperative complications can affect multiple organ systems, including the cardiovascular system, and can have an impact on the normal functioning of the heart's electrical system. One potential complication is the development of bradycardia [2].

The underlying mechanisms for sinus pauses following abdominal surgery are multifactorial. Surgical trauma and the manipulation of the abdominal cavity can activate the autonomic nervous system, resulting in an imbalance between sympathetic and parasympathetic tones. Increased parasympathetic activity, mediated by the vagus nerve, can lead to the excessive stimulation of the sinus node, causing sinus node dysfunction and resulting in sinus pauses [2].

Another possible mechanism involves the release of inflammatory mediators, such as cytokines, during surgery. Systemic inflammation can trigger an autonomic response, leading to disturbances in cardiac conduction, including sinus pauses. Additionally, pain and stress associated with surgery can activate the sympathetic nervous system, leading to an imbalance between sympathetic and parasympathetic influences on heart rate regulation [3].

Furthermore, anesthesia, pain medications, and other medications used during and after surgery can also influence the electrical conduction system of the heart, potentially leading to sinus pauses. Medications such as opioids, beta-blockers, and calcium channel blockers can have negative chronotropic and dromotropic effects, slowing down the heart rate and impairing the conduction of electrical signals within the sinus node [4].

The patient discussed in our case had sinus pauses, as well as a high-degree AV block. These are two distinct phenomena. A sinus pause refers to a temporary pause in the heart's electrical activity originating from the sinus node, while a heart block involves an interruption or a delay in the conduction of electrical signals from the atria to the ventricles [1,5,6]. Sinus pauses are often self-limiting and benign, while a heart block can have different degrees of severity depending on how long it takes for the impulse to travel to the ventricles or if some impulses do not even make it to the ventricles [1].

## Conclusions

Our case demonstrates the possibility of intra-abdominal procedures causing detrimental effects on a patient's heart. It is imperative to have a multidisciplinary approach involving surgical, medical, and critical care teams in the patient's management. Timely surgical intervention, along with aggressive resuscitation and appropriate antimicrobial therapy, is necessary for such patients. The presence of sinus pauses and complete heart block following surgery requires cardiology consultation and possibly temporary transvenous pacing. Unfortunately, in our case, despite the patient's gradual improvement in hemodynamic stability, respiratory function, and overall clinical status, she ultimately did not recover. This case highlights the importance of a multidisciplinary and individualized approach in managing critically ill surgical patients. Continual monitoring, timely interventions, and close collaboration among various specialties are vital in achieving a favorable outcome.

## References

[1] Kusumoto FM, Schoenfeld MH, Barrett C (2019). 2018 ACC/AHA/HRS guideline on the evaluation and management of patients with bradycardia and cardiac conduction delay: a report of the American College of Cardiology/American Heart Association Task Force on clinical practice guidelines and the heart. J Am Coll Cardiol.

[2] Doyle DJ, Mark PW (1990). Reflex bradycardia during surgery. Can J Anaesth.

[3] Johnston GR, Webster NR (2009). Cytokines and the immunomodulatory function of the vagus nerve. Br J Anaesth.

[4] Kim JK, Park JM, Lee CH, Kim DK (2012). Dose fentanyl injection for blunting the hemodynamic response to intubation increase the risk of reflex bradycardia during major abdominal surgery?. Korean J Anesthesiol.

[5] Dakkak W, Doukky R (2023). Sick sinus syndrome. https://pubmed.ncbi.nlm.nih.gov/29261930/.

[6] Groleau G (1986). Complete heart block. J Emerg Med.

